# Change in pre- and in-service early childhood educators’ knowledge, self-efficacy, and intentions following an e-learning course in physical activity and sedentary behaviour: a pilot study

**DOI:** 10.1186/s12889-022-12591-5

**Published:** 2022-02-07

**Authors:** Brianne A. Bruijns, Leigh M. Vanderloo, Andrew M. Johnson, Kristi B. Adamo, Shauna M. Burke, Valerie Carson, Rachel Heydon, Jennifer D. Irwin, Patti-Jean Naylor, Brian W. Timmons, Patricia Tucker

**Affiliations:** 1 Health and Rehabilitation Sciences Program, Faculty of Health Sciences, Western University, London, Ontario Canada; 2 School of Occupational Therapy, Faculty of Health Sciences, Western University, 1201 Western Road, Elborn College, Room 2547, London, ON N6G 1H1 Canada; 3 School of Health Studies, Faculty of Health Sciences, Western University, London, Ontario Canada; 4 School of Human Kinetics, Faculty of Health Sciences, University of Ottawa, Ottawa, Ontario Canada; 5 Children’s Health Research Institute, London, Ontario Canada; 6 Faculty of Kinesiology, Sport, and Recreation, University of Alberta, Edmonton, Alberta Canada; 7 Faculty of Education, Western University, London, Ontario Canada; 8 School of Exercise Science, Physical and Health Education, University of Victoria, Victoria, British Columbia Canada; 9 Child Health and Exercise Medicine Program, McMaster University, Hamilton, Ontario Canada

**Keywords:** Early childhood education, E-learning, Physical activity, Sedentary behaviour, Childcare, Self-efficacy

## Abstract

**Background:**

Early childhood educators (ECEs) are the primary daytime role models for many young children, and are responsible for facilitating physical activity (PA) opportunities and minimizing sedentary behaviour (SB) in childcare. However, they have reportedly received little related education in their pre-service training. The purpose of the Training pre-service EArly CHildhood educators in physical activity (TEACH) pilot study was to explore changes in pre- and in-service ECEs’ knowledge, self-efficacy, behavioural intention, and perceived behavioural control following the TEACH e-Learning course in PA and SB.

**Methods:**

Pre-service ECEs were purposefully recruited from three Canadian colleges, while in-service ECEs were recruited via social media. A pre-post study design was used. ECEs completed two online surveys; one prior to, and one immediately following the completion of the TEACH e-Learning course (~ 5 h). Descriptive statistics were reported, and McNemar Chi-Square tests and paired samples *t*-tests were used to examine changes in ECEs’ question-specific, and total knowledge scores, respectively. Wilcoxon Signed Ranks tests were employed to examine changes in self-efficacy, behavioural intention, and perceived behavioural control.

**Results:**

Both pre- (*n* = 32) and in-service (*n* = 121) ECEs significantly increased their total knowledge scores from pre- to post-course completion (*p* < .05*). Significant positive changes in self-efficacy (*p* < .025*), behavioural intention (*p* < .007*), and perceived behavioural control (*p* < .007*) were demonstrated by in-service ECEs following course completion, while only select composite scores within these tools were significant among pre-service ECEs.

**Conclusions:**

These findings provide preliminary evidence of the potential efficacy of the e-Learning course at improving ECEs’ knowledge, self-efficacy, behavioural intention, and perceived behavioural control to support PA and minimize SB in childcare. Following the success of the pilot study, testing the effectiveness of the TEACH e-Learning course on a larger scale, with a comparison group, is warranted prior to recommending broader dissemination of the training in pre-service ECE programs and for in-service ECE professional learning.

## Introduction

Childcare is a unique environment to promote young children’s (< 5 years) healthy physical, cognitive, and psychosocial development [1]. In developed countries, 39% of 2-year-olds, and over three-quarters of 3- and 4-year-olds, are enrolled in childcare, and spend nearly 40 h per week (~ 66% of their weekday waking hours) in these settings [2]. Young children’s movement behaviours (i.e., physical activity, sedentary behaviours, and sleep) are particularly important drivers of healthy early childhood development. Physical activity supports healthy development, such as strong bones and muscles and enhanced cognitive development [3]. Further, limiting prolonged time in sedentary pursuits, particularly screen-based behaviours, can help children avoid detrimental effects including delayed language development and decreased cognitive and psychosocial health [4, 5]. Considering young children in childcare engage in low levels of physical activity (24 min/hr), [6] and spend most of their day (66%) in sedentary behaviours, [7] interventions to support the promotion of more physically active childcare environments are critical.

To date, childcare physical activity and sedentary behaviour interventions have focused largely on: the physical environment [8, 9]; modifications to policy and practice [10–12]; and, training and support for early childhood educators (ECEs) [13–15]. The latter has proven to be essential, not only for its impact on children’s movement behaviours in childcare, [16] but also for its supportive role in facilitating successful environment, and policy and practice interventions [17, 18]. This is logical, as ECEs are highly influential in the care setting with regard to role modelling and programming physical activity and appropriate sedentary behaviours [19]. Professional learning interventions for ECEs have been noted to increase both their knowledge in and confidence to support and lead physical activity in childcare settings, [20] which seem to naturally support ECEs’ motivation and ability to utilize the environment to facilitate physical activity and carry out health-promoting changes to policy and practice – associations which are consistent with tenets of Social Cognitive Theory (SCT) [21].

Professional learning for ECEs focused on physical activity and sedentary behaviour is critical for movement behaviour interventions in childcare, as ECEs have reportedly received little education (32 and 27% of Canadian pre-service ECEs have completed courses in physical activity and screen-viewing, respectively) in these areas during their pre-service (i.e., post-secondary) training [22]. It is counterintuitive, then, to expect ECEs to carry out physical activity-promoting practices and programming in childcare settings when they often do not have the appropriate knowledge-base and know-how to support this behaviour. For example, Tucker et al.‘s [23] childcare-based intervention was designed to improve young children’s movement behaviours by providing ECEs with an evidence-based physical activity policy for 8 weeks; ECEs expressed difficulty implementing the policy components because they lacked in-depth training on how to do so [24]. Given the variability in ECEs’ educational backgrounds, it is critical that they be supported with training, both pre-service and in-service (i.e., once they begin practicing in the childcare environment), so they can confidently integrate movement (and minimize sedentary behaviour) in their daily programming and practices.

Professional learning related to children’s physical activity and sedentary behaviour has been requested by pre- and in-service ECEs themselves, [22, 25] and has been associated with increases in both ECEs’ self-efficacy (i.e., confidence to execute a particular behaviour) and their intention and perceived control over their ability to lead physical activity opportunities for the children in their care [20, 26]. SCT and the Theory of Planned Behaviour (TPB) highlight the importance of self-efficacy, behavioural intention (i.e., likelihood to perform a behaviour), and perceived behavioural control (i.e., perception of the ease or difficulty to perform a behaviour) for behaviour change, [21, 27] which are particularly important constructs to consider in these types of interventions. Specifically, self-efficacy is developed from knowledge acquisition; thus, this construct of SCT is predicted to be influenced by educational interventions [21]. Further, behavioural intention is the closest factor to human behaviour, and is often regulated by perceived behavioural control [27]; for example, ECEs may intend to program outdoor play opportunities in all weather conditions, but if their childcare centre has policies preventing outdoor play in inclement weather, this behaviour would not be within their control. Therefore, ECEs’ behavioural intention and perceived control can act as important indicators of potential behaviour change, particularly in online learning interventions. However, educator-based constructs (i.e., self-efficacy, behavioural intention, and perceived behavioural control) are infrequently measured in childcare intervention studies, and few studies explored the direct relationship between educator training and improved physical activity levels among children in childcare [28].

There has been little focus on professional learning for ECEs as an intervention uniquely (it is often coupled with prescribed physical activity programming [13]) and few researchers have explored how supplementary education in physical activity and sedentary behaviour could benefit pre-service ECEs in their post-secondary training [29]. As such, the *Training pre-service EArly CHildhood educators in physical activity* (TEACH) study was designed to to fill this gap (Tucker P, Bruijns BA, Adamo KB, Burke SM, Carson V, Heydon R, et al: Training pre-service early CHildhood educators in physical activity (TEACH): rationale and study design, submitted). The purpose of this pilot study was to examine the short-term efficacy of the TEACH e-Learning course in physical activity and sedentary behaviour on Canadian pre-service (i.e., ECE candidates enrolled in a post-secondary program) and in-service (i.e., practicing) ECEs’ related knowledge, self-efficacy, behavioural intention, and perceived behavioural control. While the TEACH e-Learning course was designed for pre-service ECEs, pilot testing in a sample of in-service ECEs was undertaken to ensure the course was relevant, informative, and helpful for real-world practice.

## Methods

Pre- and in-service ECEs were purposefully recruited to pilot test the 5-h TEACH e-Learning course in physical activity and sedentary behaviour. Expert-developed content was generated via a Delphi process [30] and the course comprised four modules developed for ECEs, which covered: introductory content on physical activity and sedentary behaviour in early childhood; the influence of the childcare environment on children’s movement behaviours, and outdoor and risky play; practical strategies to promote physical activity and minimize sedentary time among children in childcare; and, ECE-focused professional learning, resources, and a video library. For more details about the course and its development, consult the TEACH study protocol (Tucker P, Bruijns BA, Adamo KB, Burke SM, Carson V, Heydon R, et al: Training pre-service early CHildhood educators in physical activity (TEACH): rationale and study design, submitted).

### Recruitment and study procedures

From March to May 2021, three Canadian ECE programs (1-year certificate, or 2-year diploma programs) were purposefully recruited, and pre-service ECEs were eligible to participate if they were enrolled in a participating cohort. One ECE program provided in-class time for pre-service ECEs to complete the course, while the other two programs provided online (unmonitored) class time. In-service ECEs were recruited via social media advertisements (e.g., Twitter, Facebook), and were eligible to participate in the study if they were employed in a centre- or home-based childcare, preschool, or kindergarten setting. The research team also emailed Canadian and provincial/territorial childcare organizations to request that they share the study advertisement with their members. Participants were instructed to complete the e-Learning course within 2 weeks; however, accounts were not deactivated until the study closure date, which was advertised to participants via reminder emails. This pilot study was conducted in accordance with the Declaration of Helsinki and approved by the Non-Medical Research Ethics Board at Western University (REB# 116816).

### Online survey

Pre- and in-service ECEs completed an online survey (via Qualtrics; ~ 25 min) at two timepoints: (1) prior to commencing; and, (2) immediately following completion of the e-Learning course. Participants were asked to create a unique participant identification in the baseline survey to link their data to follow-up responses. The 129-item online survey comprised five sections: demographics (*n* = 12 items); knowledge (*n* = 30 items); self-efficacy (*n* = 31 items); behavioural intention (*n* = 28 items); and, perceived behavioural control (*n* = 28 items).

#### Demographics

The demographics section captured: participant age, gender, and ethnicity; province/territory; the type of ECE pre-service training program in which participants were enrolled/had completed; the number of courses in participants’ pre-service schooling (to their knowledge) that covered physical activity, outdoor play, and sedentary behaviour; their previous experience with e-Learning courses; and, their hours per week spent in moderate-to-vigorous physical activity (MVPA) and recreational screen time. Additional questions (*n* = 3) were also added to the in-service ECE baseline questionnaire, including: the type of childcare setting in which participants were employed; their years of experience; and, their past professional learning in physical activity, outdoor play, and/or sedentary behaviour.

#### Knowledge of physical activity, outdoor/risky play, and sedentary behaviour concepts

ECE knowledge was assessed via items pertaining to: *The Canadian 24-Hour Movement Guidelines for the Early Years* and movement behaviour recommendations for childcare settings (8 multiple choice items); important definitions (7 multiple choice items); appropriate ECE behaviours to support healthy movement behaviours (7 multiple choice items); and, facts about movement behaviours in childcare (8 true or false items). A composite score (out of 30) was produced.

#### Physical activity, outdoor/risky play, and sedentary behaviour self-efficacy

The valid and reliable *ECE Confidence in Outdoor Movement, Physical Activity, Sedentary and Screen behaviours* (ECE-COMPASS) questionnaire (Bruijns BA, Johnson AM, Burke SM, Tucker P: Early childhood educators’ self-efficacy to promote physical activity and outdoor play and minimize sedentary behaviour in childcare settings: A tool validation study, submitted) was administered to assess ECEs’ self-efficacy. This tool was informed by Bandura’s Guide for Creating Self-Efficacy Scales [31] and comprised of 21 task (α = 0.92; ω = 0.96; hierarchal ω = 0.60) and 10 barrier (α = 0.89; ω = 0.97; hierarchal ω = 0.79) self-efficacy items (i.e., confidence to complete a *task* [while overcoming a challenge; *barrier*]). Participants were asked to rate their confidence in their ability to perform a number of physical activity, sedentary behaviour, and outdoor play-related tasks during their childcare day on a scale from 0 (not confident at all) to 10 (completely confident). Composite scores for task and barrier self-efficacy were produced.

#### Behavioural intention and perceived behavioural control

The valid and reliable *ECE Movement Behavioural Intention and Perceived Control* (ECE-MBIPC) questionnaire [32], informed by TPB questionnaire construction recommendations, [33] and modelled after the tool employed by Gagné and Harnois, [34] was used to measure participants’ intention and perceived control to perform seven behaviours pertaining to physical activity (*n* = 3; α = 0.91), sedentary behaviour (*n* = 2; α = 0.88), and outdoor and risky play (*n* = 2; α = 0.92). Four items with a 7-point Likert scale were used to measure behavioural intention: *I have the intention to…*(1 = strongly disagree to 7 = strongly agree); *I plan to…*(1 = strongly disagree to 7 = strongly agree); *I estimate that my chances of…are* (1 = extremely unlikely to 7 = extremely likely); and, *I am going to…* (1 = extremely unlikely to 7 = extremely likely). Similarly, four items were used to measure participants’ perceived behavioural control for each of the seven abovementioned behaviours (α range = 0.88 to 0.91): *for me…would be* (1 = extremely difficult to 7 = extremely easy); *if I wanted to, I could easily…*(1 = strongly disagree to 7 = strongly agree); *it is up to me to…*(1 = strongly disagree to 7 = strongly agree); and, *I feel able to…*(1 = strongly disagree to 7 = strongly agree). Behavioural intention and perceived behavioural control composite scores for each of the seven behaviours were calculated. For behavioural intention, ω was 0.91 and hierarchal ω was 0.72. For perceived behavioural control, ω was 0.94 and hierarchal ω was 0.76.

### Data analysis

All statistical analyses were conducted in SPSS (version 27). Descriptive statistics were calculated to report participant demographics. Frequencies were generated for knowledge questionnaire responses, while means (*M*) and standard deviations (*SD*) were calculated for self-efficacy (task, barrier), behavioural intention (composite for each behaviour), and perceived behavioural control (composite for each behaviour).

To determine the efficacy of the e-Learning course with regard to increasing pre- and in-service ECEs’ knowledge, paired samples *t*-tests were run to analyze changes in *M* composite scores, and McNemar chi square tests were conducted for individual questions. Considering the self-efficacy, behavioural intention, and perceived behavioural control data were non-normally distributed (Shapiro-Wilk = 0.86; *p* < .000*), Wilcoxon Signed Ranks Tests were used. Bonferroni corrections were performed to account for familywise error within each set of multiple comparisons.

## Results

Fifty-one pre-service ECEs completed the baseline survey (from 65 invited; 78.5% response rate) and 36 completed the follow-up survey (32 retained for analysis [i.e., participant ID matched baseline survey]; 59.3% retention from baseline).^1^ From the 274 in-service ECEs that were recruited at baseline, 133 completed the follow-up survey, and 121 were retained for analysis (42.3% retention from baseline).^2^

### Participant demographics

Pre-service ECEs were from Ontario (34.4%), Alberta (18.8%), and the Northwest Territories (21.9%). Participants were female (93.8%), 26.7 years old (*SD* = 6.9), and most were South Asian (28.1%%) or First Nations/Inuit/Métis (28.1%), and enrolled in an early childhood education diploma program (93.8%). The vast majority of participants self-reported that their program offered at least one course covering content relating to physical activity (100.0%), sedentary behaviours (87.7%), and outdoor and/or risky play (91.9%). Most participants (65.6%) had previous experience with e-Learning courses/workshops. A minority of pre-service ECEs self-reported to meet the MVPA guideline (150+ min/week; 31.3%) or the recreational screen time guideline (< 3 h/day; 37.5%) outlined in the *Canadian 24-Hour Movement Guidelines for Adults* [35].

In-service ECEs represented seven Canadian provinces/territories. The average age of in-service ECEs was 37.1 years (*SD* = 9.5), and most were female (99.2%), Caucasian (66.1%), employed in a centre-based childcare setting (62.5%), and had an average of 10.9 (*SD* = 8.8) years of experience as an ECE. Reflecting on their pre-service training, 67.8% of in-service ECEs completed a diploma program, and many reported having taken at least one course covering content in physical activity (81%), sedentary behaviours (47.9%), and outdoor and/or risky play (77.6%). A number of ECEs also reported having completed professional learning in physical activity (38.0%), sedentary behaviour (16.5%), and outdoor and/or risky play (56.2%), and 70.2% had previous experience with e-Learning courses/workshops. Just over a quarter of ECEs (28.1%) self-reported to meet the MVPA guideline within the *Canadian 24-Hour Movement Guidelines for Adults,* [35] while most ECEs (69.4%) met the recreational screen time guideline. See Table 1 for full participant demographics.

**Table 1:** Pre- and in-service early childhood educators’ demographic information

Variable	Pre-Service (***N*** = 32)		In-Service (***N*** = 121)		Variable	Pre-Service (***N*** = 32)		In-Service (***N*** = 121)	
	***n***	%	***n***	%		***n***	%	***n***	%
**Age (*M, SD*)**	26.7	6.9	37.1	9.5	**Current/Past ECE Program Type**				
**Gender**					Certificate	2	6.3	12	9.9
Female	30	93.8	120	99.2	Diploma	30	93.8	82	67.8
Male	2	6.3	1	.8	Bachelor’s Degree	–	–	18	14.9
**Ethnicity**					Graduate Degree	–	–	4	3.3
Caucasian	5	15.6	80	66.1	Other	–	–	5	4.1
African Canadian	1	3.1	2	1.7	**Years of ECE Experience (*M, SD*)**	–	–	10.9	8.8
South Asian	9	28.1	10	8.3	**ECE Program Courses in Physical Activity**^**b**^				
East Asian	4	12.5	11	9.1	No courses	–	–	23	19.0
Southeast Asian	2	6.3	3	2.5	1 course	3	9.4	64	52.9
Middle Eastern	–	–	3	2.5	2 courses	11	34.4	22	18.2
First Nations/Inuit/Métis	9	28.1	1	.8	3+ courses	18	56.2	12	9.9
Latin Canadian	1	3.1	4	3.3	**ECE Program Courses in Sedentary Behaviour**^**b**^				
Other	1	3.1	4	3.3	No courses	4	12.5	63	52.1
Prefer not to answer	–	–	3	2.5	1 course	10	31.3	37	30.6
**Province/Territory**					2 courses	2	6.3	10	8.3
Alberta	6	18.8	24	20.0	3+ courses	16	50.1	11	9.0
British Columbia	–	–	16	13.3	**ECE Program Courses in Outdoor and Risky Play**^**b**^				
Manitoba	–	–	7	5.8	No courses	2	6.3	27	22.3
Newfoundland & Labrador	–	–	5	4.2	1 course	2	6.3	62	51.2
Northwest Territories	7	21.9	4	3.3	2 courses	7	21.9	15	12.4
Ontario	11	34.4	61	50.8	3+ courses	21	63.7	17	14.0
Saskatchewan	–	–	3	2.5	**Childcare Type**				
**Meeting the Adult Physical Activity Guideline**^**a**^					Centre-based childcare	–	–	75	62.5
Yes	10	31.3	34	28.1	Home-based childcare	–	–	11	9.2
No	22	68.8	87	71.9	Kindergarten	–	–	18	15.0
**Meeting the Adult Screen Time Guideline**^**a**^					Preschool	–	–	16	13.3
Yes	12	37.5	84	69.4	**Professional Development**				
No	20	62.5	37	30.6	Physical Activity	–	–	46	38.0
**Previous e-Learning Experience**					Sedentary Behaviour	–	–	20	16.5
Yes	21	65.6	85	70.2	Outdoor/Risky Play	–	–	68	56.2
No	11	34.4	36	29.8	None	–	–	41	33.9

*Notes.* ECE = Early Childhood Education; – = not applicable; ^a^ 150 min/week of moderate-to-vigorous physical activity and < 3 h/day of recreational screen time as per the *Canadian 24-Hour Movement Guidelines for Adults* (CSEP, 2020); ^b^ Self-reported courses in program

### Knowledge of physical activity and sedentary behaviour concepts

**Fig. 1:**
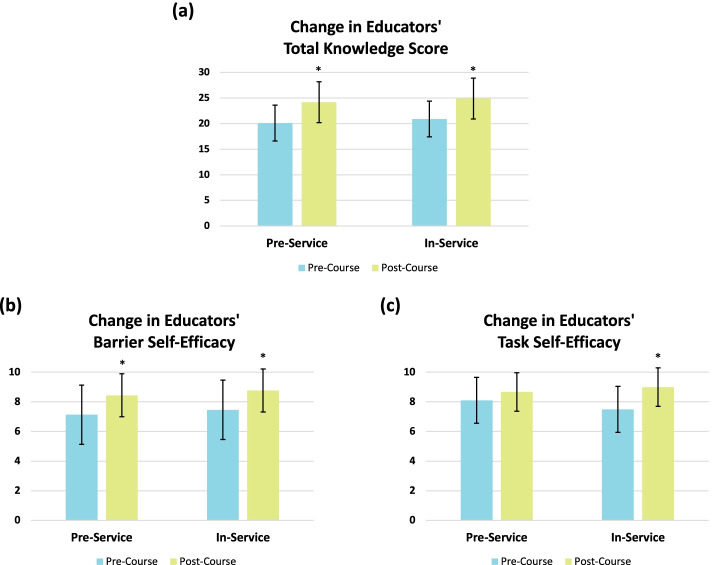
**a** Change in pre- and in-service early childhood educators’ (ECEs) total knowledge score (out of 30) from pre-course to post-course (* = significant [*p* < .05]); (**b**) Change in pre- and in-service ECEs’ barrier self-efficacy from pre-course to post-course (* = significant [*p* < .025]); (**c**) Change in ECEs’ task self-efficacy from pre-course to post-course (* = significant [*p* < .025])

There were significant improvements in pre-service participants’ total knowledge score from pre- to post-course (Fig. 1a). While item-specific answers trended in the expected direction (i.e., increase in percentage of correct responses), insufficient cell sizes prevented item-by-item analysis. Similarly, in-service ECEs’ total knowledge score also increased significantly from pre- to post-course (Fig. 1a). Of note, ECEs significantly increased their knowledge of the physical activity and screen time guidelines within the *Canadian 24-Hour Movement Guidelines for the Early Years*. For example, when asked to select the appropriate screen time guideline for a 3-year-old, only 11.6% of ECEs indicated the correct time limit pre-course, whereas 61.9% of ECEs selected the correct answer after completing the course (*X*^*2*^ [117] = 50.21, *p* = .000). See Table 2 for further item-specific data.

**Table 2:**
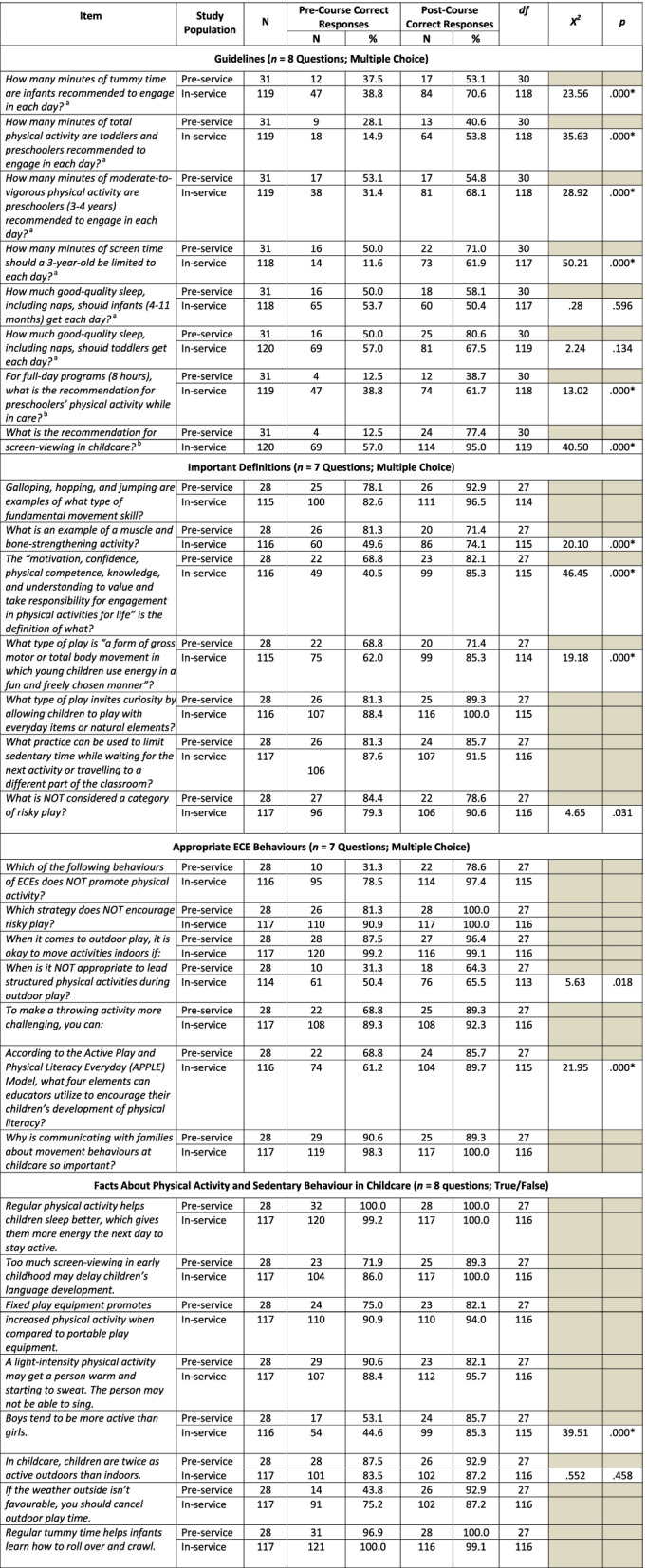
Change in early childhood educators’ knowledge following the e-learning course

*Note.* ^a^As per the *Canadian 24-Hour Movement Guidelines for the Early Years* (CSEP, 2017); ^b^ As per research-based recommendations presented in the e-Learning course; McNemar Chi Square Tests were between early childhood educators’ pre- and post-course self-efficacy ratings; Shaded box = McNemar statistics could not be computed due to insufficient cell size and/or item difficulty; * = significant <.000 after adjusting for multiple comparison bias

### Physical activity and sedentary behaviour self-efficacy

There was a significant change in pre-service ECEs’ barrier self-efficacy from pre- to post-course (Fig. 1b), but not in their task self-efficacy (Fig. 1c). For in-service ECEs, there was a significant positive change in both their task and barrier self-efficacy from pre- to post-course completion (Fig. 1 b and c).

### Behavioural intention and perceived behavioural control

Pre-service ECEs’ behavioural intention to “*promote outdoor play*” and “*avoid screen use during childcare*” increased significantly from pre- (*M* = 5.70 [*SD* = 1.44] vs. *M* = 5.58 [*SD* = 1.35], *Z* = 3.227, *p* = .001, respectively) to post-course (*M* = 6.58 [*SD* = .70] vs. *M* = 6.61 [*SD* = .78], *Z* = − 2.921, *p* = .003, respectively). Further, pre-service ECEs’ perceived behavioural control to “*engage children in my care in at least 120 min/day of physical activity*” and “*avoid screen use during childcare*” increased significantly from pre-course (*M* = 5.88 [*SD* = .78] vs. *M* = 5.92 [*SD* = .87], *Z* = − 2.858, *p* = .004, respectively) to post-course completion (*M* = 6.32 [*SD* = .73] vs. *M* = 6.46 [*SD* = .74], *Z* = − 2.958, *p* = .003, respectively). However, there were no significant differences in behavioural intention or perceived control for any of the remaining behaviours (*p* > .007). In-service ECEs significantly increased behavioural intention and perceived behavioural control across all seven behaviours (*p* < .007; Table 3). See Table 3 for item-specific analyses.

**Table 3:** Change in early childhood educators’ behavioural intention and perceived behavioural control following the e-learning course

Item	Study Population	N	Pre-Course		Post-Course		+ve Rank (N)	-ve Rank (N)	***Z***	***p***
			***M*** (***SD***)	Median (IQR)	***M*** (***SD***)	Median (IQR)				
**Behavioural Intention (*n*** **= 7 items)**										
Engage children in my care in at least 120 min/day of physical activity	Pre-service	26	6.06 (.90)	6.0 (5.75–6.75)	6.55 (.57)	6.75 (6.25–7.0)	12	7	−1.757	.079
	In-service	113	6.05 (.95)	6.25 (5.5–7.0)	6.42 (.69)	6.5 (6.0–7.0)	62	19	−4.782	.000*
Promote children’s development of physical literacy by incorporating fundamental movement skills	Pre-service	26	6.26 (.68)	6.25 (6.0–7.0)	6.59 (.62)	7.0 (6.25–7.0)	11	8	−1.719	.086
	In-service	112	6.22 (.75)	6.25 (5.88–7.0)	6.53 (.60)	6.75 (6.0–7.0)	58	21	−4.469	.000*
Be a good role model for children’s physical activity by participating in movement-based activities	Pre-service	24	6.42 (.70)	6.75 (6.0–7.0)	6.63 (.67)	7.0 (6.38–7.0)	10	5	−.799	.424
	In-service	112	6.27 (.86)	6.5 (6.0–7.0)	6.59 (.72)	7.0 (6.25–7.0)	57	11	−5.119	.000*
Promote outdoor play during all seasons and weather conditions	Pre-service	26	5.70 (1.44)	6.0 (4.5–7.0)	6.58 (.70)	7.0 (6.25–7.0)	16	2	−3.227	.001*
	In-service	109	6.28 (.89)	6.5 (6.0–7.0)	6.61 (.49)	6.75 (6.25–7.0)	48	25	−4.143	.000*
Lead opportunities for outdoor risky play for children in my care	Pre-service	26	5.73 (1.50)	6.0 (5.5–7.0)	6.42 (.75)	6.75 (6.0–7.0)	14	6	−2.037	.042
	In-service	111	5.74 (1.31)	6.0 (5.25–6.75)	6.16 (1.02)	6.25 (6.0–7.0)	62	19	−4.226	.000*
Minimize long periods of sedentary time (> 60 min) for children in my care	Pre-service	25	5.95 (1.10)	6.0 (5.63–7.0)	6.50 (.74)	6.75 (6.25–7.0)	11	5	−1.997	.046
	In-service	111	6.14 (1.01)	6.5 (5.75–7.0)	6.53 (.61)	6.75 (6.0–7.0)	61	18	−4.399	.000*
Avoid children’s use of screen-based technology during childcare hours	Pre-service	26	5.58 (1.35)	6.0 (4.25–7.0)	6.61 (.78)	7.0 (6.5–7.0)	16	3	−2.921	.003*
	In-service	112	6.35 (1.11)	7.0 (6.0–7.0)	6.73 (.61)	7.0 (6.75–7.0)	41	13	−4.263	.000*
**Perceived Behavioural Control (*n*** **= 7 items)**										
Engage children in my care in at least 120 min/day of physical activity	Pre-service	27	5.88 (.78)	6.0 (5.31–6.69)	6.32 (.73)	6.5 (6.0–7.0)	19	4	−2.858	.004*
	In-service	112	5.69 (1.22)	6.0 (4.88–6.75)	6.16 (1.14)	6.5 (5.75–7.0)	64	20	−4.947	.000*
Promote children’s development of physical literacy by incorporating fundamental movement skills	Pre-service	28	6.01 (.65)	6.0 (5.75–6.69)	6.38 (.75)	6.75 (6.0–7.0)	19	5	−2.465	.014
	In-service	109	5.99 (.97)	6.0 (5.5–6.75)	6.46 (.62)	6.75 (6.0–7.0)	61	20	−5.268	.000*
Be a good role model for children’s physical activity by participating in movement-based activities	Pre-service	27	6.32 (.60)	6.25 (6.0–7.0)	6.49 (.71)	6.75 (6.0–7.0)	12	5	−1.839	.066
	In-service	106	6.16 (.88)	6.5 (5.75–7.0)	6.50 (.84)	7.0 (6.0–7.0)	56	16	−4.734	.000*
Promote outdoor play during all seasons and weather conditions	Pre-service	26	5.76 (1.17)	6.0 (5.25–6.75)	6.33 (.84)	6.75 (6.0–7.0)	15	5	−2.692	.007
	In-service	111	6.03 (.94)	6.25 (5.5–7.0)	6.37 (.72)	6.63 (6.0–7.0)	57	22	−3.790	.000*
Lead opportunities for outdoor risky play for children in my care	Pre-service	28	5.84 (1.12)	6.0 (5.56–6.75)	6.27 (.92)	6.88 (6.0–7.0)	16	5	−1.916	.055
	In-service	107	5.42 (1.38)	5.75 (4.5–6.56)	5.88 (1.21)	6.0 (5.5–6.75)	67	20	−4.736	.000*
Minimize long periods of sedentary time (> 60 min) for children in my care	Pre-service	28	5.91 (.88)	5.88 (5.25–6.75)	6.35 (.81)	6.88 (6.0–7.0)	14	5	−2.339	.019
	In-service	110	6.00 (.95)	6.25 (5.5–6.75)	6.41 (.78)	6.75 (6.0–7.0)	65	20	−4.995	.000*
Avoid children’s use of screen-based technology during childcare hours	Pre-service	28	5.92 (.87)	6.0 (5.25–6.75)	6.46 (.74)	6.88 (6.0–7.0)	17	6	−2.958	.003*
	In-service	109	6.25 (1.13)	6.75 (5.75–7.0)	6.62 (.72)	7.0 (6.5–7.0)	50	18	−4.157	.000*

*Note. M* = mean; *SD* = standard deviation; IQR = interquartile range; Behavioural intention and perceived behavioural control were scored on a 7-point Likert scale using 4 questions each (composite scores across these 4 questions are presented); Wilcoxon Signed Ranks Tests were between early childhood educators’ pre- and post-course behavioural intention and perceived behavioural control ratings; Where positive and negative rank N values do not equal sample N, remaining participants tied their baseline score; * = significant after adjusting for multiple comparison bias (alpha compared at .0071)

## Discussion

Given ECEs have been noted to largely influence movement affordances in childcare settings, [36] ensuring they have the understanding, confidence, and motivation to facilitate physical activity opportunities in these settings is important. To our knowledge, this is the first study to examine the short-term effect of an e-Learning course in physical activity and sedentary behaviour on both pre- and in-service ECEs’ knowledge, self-efficacy, behavioural intention, and perceived behavioural control to support physical activity and minimize sedentary behaviour in childcare. After taking the course, both pre- and in-service ECEs demonstrated significant positive changes in their knowledge and self-efficacy regarding physical activity, sedentary behaviour, and outdoor play in childcare settings. Their intention and perceived control to promote healthy levels of physical activity and appropriate sedentary behaviour also increased following training. A number of these findings are discussed below.

As noted above, both pre- and in-service ECEs significantly increased their total knowledge of physical activity and sedentary behaviour. These improvements could largely be attributed to increased scores in the *Guidelines* and *Important Definitions* sections of the questionnaire. Of note, very few ECEs demonstrated an understanding of the physical activity and screen-viewing recommendations within the *24-Hour Movement Guidelines for the Early Years* prior to taking the e-Learning course. This is consistent with previous work by Bruijns and colleagues, [17] which showed that less than 20% and 13% of ECEs (*n* = 83) correctly recalled physical activity and screen-viewing guidelines, respectively, prior to participating in training. More positively, findings from the present study showed that in-service ECEs’ guideline recollection approached 100% for some items following the e-Learning course, indicating that participants were able to learn this content effectively via e-Learning. Significant increases were also observed for the in-service ECEs who provided the correct responses for questions pertaining to physical literacy, active play, and muscle and bone-strengthening activities definitions. Our baseline finding related to physical literacy and subsequent improvements aligns with the findings of Foulkes and colleagues, [37] who found early care providers were not aware of the meaning of the term ‘physical literacy’. It is clear that ECEs need additional training in physical activity domains to both understand the importance of being active in a variety of ways and how to integrate active play experiences into early learning settings.

In addition to marked increases in pre- and in-service ECEs’ knowledge, the e-Learning course was also associated with a significant increase in ECEs’ self-efficacy. This finding speaks to the well-rounded nature of the e-Learning course, as previous professional learning studies with ECEs have typically focused only on children’s physical activity, [38–40] with sedentary behaviour often left out. By including sedentary behaviour content and placing focus on the importance of outdoor play in facilitating physical activity among children in childcare, the ECEs in our study appear to have gained confidence in these other domains as well. Similarly, Hassani et al. [20] measured Canadian ECEs’ (*n* = 1819) confidence following a professional learning intervention in healthy eating and physical activity (which also included content on sedentary behaviour), and found that ECEs demonstrated significant increases in both physical activity and sedentary behaviour-related confidence. As such, supporting ECEs’ self-efficacy development via professional learning is a useful tool that can increase the likelihood that they will incorporate movement-based programming, while satisfying their request for additional training in these domains.

Not only did ECEs show improvements in their knowledge and self-efficacy scores, but behavioural intention and perceived behavioural control relating to physical activity and sedentary behaviour also increased, consistent with previous literature [26]. Bai and colleagues [26] implemented both a nature play and a fundamental movement skill professional learning intervention for Australian ECEs (*n* = 84 and *n* = 64, respectively), and observed significant increases in self-efficacy, intention, and perceived behavioural control for promoting physical activity. In accordance with the TPB, [27] when ECEs exhibit greater intention to promote active childcare settings and better ability to control their practices and programming, behaviour change is expected. The intersection of these psychosocial variables is likely to influence children’s physical activity levels in childcare, [34] which is important to consider when designing childcare-based intervention studies. As such, fostering ECEs’ own knowledge, confidence, intentions, and perceived control is an efficacious way to promote sustainable change in the childcare setting with respect to movement opportunities.

### Research implications and future directions

The findings from this pilot study are important for public health researchers in the early years population. Specifically, the comprehensiveness of the e-Learning course itself, which included content on physical activity, sedentary behaviour, and outdoor and risky play, lends itself to be applicable to childcare providers both within and outside of Canada, as the course was not designed for a specific program or intervention, but rather to provide general training in these areas. The preliminary efficacy of the e-Learning course at increasing ECEs’ physical activity and sedentary behaviour-related knowledge, self-efficacy, behavioural intention, and perceived behavioural control is encouraging for the use of this training to address public health issues, such as physical inactivity in childcare settings, by ensuring ECEs understand how to and are confident in promoting healthy physical activity and sedentary behaviour in early learning environments. Moreover, the virtual nature of the course increases the potential for population-level reach, and only simple modifications would be needed to tailor it for other settings. Future research in this field should explore whether ECEs’ knowledge, self-efficacy, behavioural intention, and perceived behavioural control (uniquely or in combination) are important drivers of young children’s physical activity in the childcare setting.

### Strengths and limitations

While this pilot study has many strengths including its diverse Canadian sample, inclusion of both pre- and in-service ECEs, and the high response rate within the context of online surveys, there are also limitations which must be highlighted. First, as this was a pilot study, findings should be interpreted with caution given there was no control group against which to compare intervention samples. Second, the small sample size of the pre-service ECEs, due to logistical issues with implementation in post-secondary settings during the COVID-19 pandemic, lacked the power needed to demonstrate complete intervention efficacy in this population. Further, the low retention of in-service ECEs (~ 40%), as compared to pre-service ECEs (~ 60%), is important to acknowledge, as in-service ECEs retained for analysis differed on select demographic variables from those lost to follow-up. While these differences in retention may have been attributed to the differential recruitment and implementation methods in these study populations, as well as the burden of the COVID-19 pandemic on in-service ECEs’ time to partake in professional learning, it is possible these differences may have impacted the study findings. Third, the knowledge questionnaire was not a validated instrument, as it was created based on the specific e-Learning course content which was unique to this study. As such, while face validity was achieved through expert review and consensus, measures of knowledge in this study may not be generalizable to other research with this population. Further, lack of sufficient cell sizes and item difficulty within the questionnaire limited the analyses that could be conducted with these data. Finally, given the self-reported nature of the online survey, social desirability bias may have been at play, as ECEs may have felt that more positive responses (i.e., rating themselves as more confident or intentional) were expected of someone in their profession. Despite these limitations, we found significance in a study that was underpowered to do so; as such, it is predicted that scale-up of this pilot study with a more robust sample and a comparison group is likely to demonstrate effectiveness within this population.

## Conclusion

Utilizing e-Learning to train both pre- and in-service ECEs in physical activity and sedentary behaviour may be an effective strategy to ensure they are competent, confident, and motivated to promote physical activity and minimize sedentary behaviours in childcare. Given the current paucity of educator-focused outcome measures in early years physical activity literature, [28] this study’s findings provide preliminary evidence that educator-based factors such as knowledge, self-efficacy, and behavioural intention and perceived control may play an important role in how physical activity, sedentary behaviour, and outdoor play are valued and facilitated by ECEs in childcare programming. While additional testing with a more robust sample and comparison group is needed before specific recommendations can be made, the potential reach and public health impact of e-Learning in physical activity and sedentary behaviour for ECEs is vast.

## Data Availability

The datasets generated and/or analyzed during this current study are not publicly available due to ethical restrictions but are available from the corresponding author on reasonable request.

## Abbreviations

ECE: Early childhood education
ECE COMPASS: Early Childhood Educator Confidence in Outdoor Movement, Physical Activity, Sedentary and Screen behaviours
ECE-MBIPC: ECE Movement Behavioural Intention and Perceived Control
SCT: Social Cognitive Theory
MVPA: Moderate-to-vigorous physical activity
TPB: Theory of Planned Behaviour

## Footnotes

1 Pre-service ECEs retained for analysis were significantly younger, and reported to: have taken more courses in physical activity; have experience with e-Learning workshops; and, not meet the physical activity and screen time guidelines within the *Canadian 24-Hour Movement Guidelines for Adults*, than those lost to follow-up (*p* < 0.05).

2 In-service ECEs retained for analysis were significantly more likely to have completed a diploma program and meet the screen time guideline within the *Canadian 24-Hour Movement Guidelines for Adults*, than those lost to follow-up (*p* < 0.05).

## References

[1] Goldfield GS, Harvey A, Grattan K, Adamo KB (2012). Physical activity promotion in the preschool years: a critical period to intervene. Int J Environ Res Public Health.

[2] OECD (2021). Enrolment rate in early childhood education (indicator).

[3] Carson V, Lee E-Y, Hewitt L, Jennings C, Hunter S, Kuzik N (2017). Systematic review of the relationships between physical activity and health indicators in the early years (0-4 years). BMC Public Health.

[4] Carson V, Rahman AA, Wiebe SA. Associations of subjectively and objectively measured sedentary behaviour and physical activity with cognitive development in the early years. Ment Health Phys Act. 2017;13:1–8. Available from: 10.1016/j.mhpa.2017.05.003.

[5] Leblanc AG, Spence JC, Carson V, Connor Gorber S, Dillman C, Janssen I (2012). Systematic review of sedentary behaviour and health indicators in the early years (aged 0-4 years). Appl Physiol Nutr Metab.

[6] Vanderloo LM, Tucker P, Johnson AM, Van Zandvoort MM, Burke SM, Irwin JD (2014). The influence of Centre-based childcare on preschoolers’ physical activity levels: a cross-sectional study. Int J Environ Res Public Health.

[7] Tucker P, Vanderloo LM, Burke SM, Irwin JD, Johnson AM (2015). Prevalence and influences of preschoolers’ sedentary behaviours in early learning centres: a cross-sectional study. BMC Pediatr.

[8] Cosco NG, Moore RC, Smith WR (2014). Childcare outdoor renovation as a built environment health promotion strategy: evaluating the preventing obesity by design intervention. Sci Lifestyle Chang.

[9] Robinson JC, Temple ML, Duck A, Klamm M (2019). Feasibility and effectiveness of two built environmental interventions on physical activity among 3–5-year-old preschoolers. J Spec Pediatr Nurs.

[10] Carson V, Clark D, Ogden N, Harber V, Kuzik N (2015). Short-term influence of revised provincial accreditation standards on physical activity, sedentary behaviour, and weight status in Alberta, Canada child care centres. Early Child Educ J.

[11] Finch M, Wolfenden L, Edenden D, Falkiner M, Pond N, Hardy L (2012). Impact of a population based intervention to increase the adoption of multiple physical activity practices in Centre based childcare services: a quasi experimental, effectiveness study. Int J Behav Nutr Phys Act.

[12] Erinosho T, Mason K, Morris E, Schwartz E Ward D. Evaluating the presence of formal nutrition and physical activity policies in childcare centres in North Carolina. FASEB J. 2014;28(1 SUPPL. 1). Available from: 10.1096/fasebj.28.1_supplement.132.1.

[13] Green AM, Mihrshahi S, Innes-Hughes C, O’Hara BJ, McGill B, Rissel C. Implementation of an early childhood healthy eating and physical activity program in New South Wales, Australia: Munch & Move. Front Public Heal. 2020;8:34 [cited 2021 May 27] Available from: 10.3389/fpubh.2020.00034. 10.3389/fpubh.2020.00034 PMC7047441 32154206

[14] Pate RR, Brown WH, Pfeiffer KA, Howie EK, Saunders RP, Addy CL (2016). An intervention to increase physical activity in children: a randomized controlled trial with 4-year-olds in preschools. Am J Prev Med.

[15] Tucker P, Vanderloo LM, Johnson AM, Burke SM, Irwin JD, Gaston A (2017). Impact of the supporting physical activity in the childcare environment (SPACE) intervention on preschoolers’ physical activity levels and sedentary time: a single-blind cluster randomized controlled trial. Int J Behav Nutr Phys Act.

[16] Trost SG, Ward DS, Senso M (2010). Effects of child care policy and environment on physical activity. Med Sci Sports Exerc.

[17] Bruijns BA, Johnson AM, Irwin JD, Burke SM, Driediger M, Vanderloo LM, et al. Training may enhance early childhood educators’ self-efficacy to Lead physical activity in childcare. BMC Public Health. 2021;21(1):386. Available from: 10.1186/s12889-021-10400-z 10.1186/s12889-021-10400-z PMC7893737 33607984

[18] Howie EK, Brewer A, Brown WH, Saunders R, Pate RR (2016). Systematic dissemination of a preschool physical activity intervention to the control preschools.

[19] Robinson LE, Wadsworth DD, Peoples CM (2012). Correlates of school-day physical activity in preschool students. Res Q Exerc Sport.

[20] Hassani K, Buckler EJ, Nzunga JM, Fakih S, Scarr J, Mâsse LC, et al. Implementing Appetite to Play at scale in British Columbia : Evaluation of a Capacity - Building Intervention to Promote Physical Activity in the Early Years; 2020. p. 1–19. Available from: 10.3390/ijerph17041132. 10.3390/ijerph17041132 PMC7068589 32053916

[21] Health BA (2004). Promotion by Social Cognitive Means. Heal Educ Behav.

[22] Bruijns BA, Adamo KB, Burke SM, Carson V, Irwin JD, Naylor P-J (2019). Exploring the physical activity and screen-viewing-related knowledge, training, and self-efficacy of early childhood education candidates. BMC Pediatr.

[23] Tucker P, Driediger M, Vanderloo LM, Burke SM, Irwin JD, Johnson AM, et al. Exploring the feasibility and effectiveness of a Childcare PhysicaL ActivitY (PLAY) policy: Rationale and protocol for a pilot, cluster-randomized controlled trial. Int J Environ Res Public Health. 2019;16(22):4400. Available from: 10.3390/ijerph16224400 10.3390/ijerph16224400 PMC6888608 31717931

[24] Szpunar M, Johnson AM, Driediger MV, Burke SM, Irwin JD, Shelley J, et al. Implementation adherence and perspectives of the childcare PhysicaL ActivitY (PLAY) policy: A process evaluation. Health Educ Behav. 2021(0):1-12. Available from: 10.1177/1090198121996285. 10.1177/1090198121996285 PMC8892048 33749362

[25] van Zandvoort M, Tucker P, Irwin JD, Burke SM (2010). Physical activity at daycare: issues, challenges and perspectives. Early Years.

[26] Bai P, Thornton A, Lester L, Schipperijn J, Trapp G, Boruff B (2020). Nature play and fundamental movement skills training programs improve childcare educator supportive physical activity behaviour. Int J Environ Res Public Health.

[27] Ajzen I (1991). The theory of planned Behaviour. Vol. 50, Organizational behaviour and human decision processes.

[28] Peden ME, Okely AD, Eady MJ, Jones RA (2018). What is the impact of professional learning on physical activity interventions among preschool children? A systematic review. Clin Obes.

[29] Altunsöz IH (2015). Early childhood education majors’ self-efficacy for teaching fundamental motor skills. Percept Mot Skills.

[30] Bruijns BA, Johnson AM, Tucker P (2020). Content development for a physical activity and sedentary behaviour e-learning module for early childhood education students: a Delphi study. BMC Public Health.

[31] Bandura A (2006). Guide for constructing self-efficacy scales. Self-efficacy Beliefs of Adolescents.

[32] Bruijns BA, Johnson AM, Burke SM, Tucker P. Validation of a physical activity, sedentary behaviour, and outdoor play behavioural intention and perceived behavioural control tool for early childhood educators. Article accepted for publication in Early Child Educ J. 2022.

[33] Ajzen I (2013). Theory of Planned Behaviour Questionnaire.

[34] Gagné C, Harnois I (2013). The contribution of psychosocial variables in explaining preschoolers’ physical activity. Health Psychol.

[35] Canadian Society for Exercise Physiology. Canadian 24-Hour Movement Guidelines for Adults ages 18–64 years. 2020. Available from: https://csepguidelines.ca/guidelines/adults-18-64/

[36] Copeland KA, Kendeigh CA, Saelens BE, Kalkwarf HJ, Sherman SN (2012). Physical activity in child-care centres: do teachers hold the key to the playground?. Health Educ Res.

[37] Foulkes JD, Foweather L, Fairclough SJ, Knowles Z. “I Wasn’t sure what it meant to be honest”—formative research towards a physical literacy intervention for preschoolers. Children. 2020;7(7):76.[cited 2021 Jun 11] Available from: 10.3390/children7070076 10.3390/children7070076 PMC7401874 32668611

[38] Adamo KB, Wasenius NS, Grattan KP, Harvey ALJ, Naylor P-J, Barrowman NJ (2017). Effects of a preschool intervention on physical activity and body composition. J Pediatr.

[39] Hoffman JA, Schmidt EM, Castaneda-Sceppa C, Hillman CH (2019). The theoretical foundation, fidelity, feasibility, and acceptability of a teacher training to promote physical activity among preschoolers in child care: a pilot study. Prev Med Reports.

[40] Pfeiffer KA, Saunders R, Brown WH, Dowda M, Addy CL, Pate RR (2013). Study of health and activity in preschool environments (SHAPES): study protocol for a randomized trial evaluating a multi-comonent physical intervention in preschool children. BMC Public Health.

